# Targeting Chronic Inflammation of the Digestive System in Cancer Prevention: Modulators of the Bioactive Sphingolipid Sphingosine-1-Phosphate Pathway

**DOI:** 10.3390/cancers14030535

**Published:** 2022-01-21

**Authors:** Eileen M. McGowan, Yiguang Lin, Size Chen

**Affiliations:** 1 Central Laboratory, The First Affiliated Hospital of Guangdong Pharmaceutical University, Guangzhou 510080, China; Yiguang.Lin@uts.edu.au (Y.L.); chensize@gdpu.edu.cn (S.C.); 2 Guangdong Provincial Engineering Research Center for Esophageal Cancer Precise Therapy, The First Affiliated Hospital of Guangdong Pharmaceutical University, Guangzhou 510080, China; 3 School of Life Sciences, University of Technology Sydney, Broadway, Sydney, NSW 2007, Australia

**Keywords:** sphingosine-1-phosphate (S1P), sphingosine kinase (SphK), gastrointestinal cancers, digestive system, S1P modulators, inflammation, immunotherapy

## Abstract

**Simple Summary:**

Obesity, an ongoing global pandemic, is a major contributor to inflammation and cancers of the digestive system. High saturated fat diets and being overweight are associated with chronic inflammation and increased cancer risk. Signalling molecules made from saturated fats known as bioactive sphingolipids play essential roles in healthy gastrointestinal immunity. In excess, these sphingolipid molecules can compromise our immune system leading to chronic, low-grade inflammation within the digestive system preceding many metabolic diseases including cancer. Sphingosine-1-phosphate is a bioactive sphingolipid and, in excess, contributes to chronic inflammation. Drugs that block sphingosine-1-phosphate activity have the potential to prevent chronic inflammation and reduce gastrointestinal cancer risk. We review how disruption of the sphingosine-1-phosphate pathway contributes to gastrointestinal inflammation and cancer. We also discuss the use of modulators of the sphingospine-1-phosphate pathway in clinical trials and in the clinic as therapeutics for inflammatory gastrointestinal diseases with the benefit of reducing cancer risk.

**Abstract:**

Incidence of gastrointestinal (GI) cancers is increasing, and late-stage diagnosis makes these cancers difficult to treat. Chronic and low-grade inflammation are recognized risks for most GI cancers. The GI mucosal immune system maintains healthy homeostasis and signalling molecules made from saturated fats, bioactive sphingolipids, play essential roles in healthy GI immunity. Sphingosine-1-phosphate (S1P), a bioactive sphingolipid, is a key mediator in a balanced GI immune response. Disruption in the S1P pathway underlies systemic chronic metabolic inflammatory disorders, including diabetes and GI cancers, providing a strong rationale for using modulators of the S1P pathway to treat pathological inflammation. Here, we discuss the effects of bioactive sphingolipids in immune homeostasis with a focus on S1P in chronic low-grade inflammation associated with increased risk of GI carcinogenesis. Contemporary information on S1P signalling involvement in cancers of the digestive system, from top to bottom, is reviewed. Further, we discuss the use of novel S1P receptor modulators currently in clinical trials and their potential as first-line drugs in the clinic for chronic inflammatory diseases. Recently, ozanimod (Zeposia^TM^) and etrasimod have been approved for clinical use to treat ulcerative colitis and eosinophilic oesophagitis, respectively, which may have longer term benefits in reducing risk of GI cancers.

## 1. Introduction

Cancers of the digestive system (gastro-intestinal (GI) and associated GI cancers), in general, have low overall 5-year survival rates mainly due to their late-stage diagnosis [1]. Incidence and mortality rates of all GI tract cancer patients are predicted to increase significantly over the next 20 years (Table 1) [2,3,4,5].

For over a century, the association between chronic inflammation and GI cancers has been recognised [6]. The hostile ever-changing GI microenvironments, the constant renewal of the epithelial lining and the interaction of the epithelial, stromal, and immune cells, and the relentless onslaught of pathogens in this nutrient-rich environment, makes the digestive system particularly vulnerable to inflammation [7]. Inflammatory bowel diseases (IBDs), such as ulcerative colitis (UC) and Crohn’s disease (CD), arise from prolonged low-grade systemic inflammation in the digestive tract, with a high risk of cancer development within sites of chronic inflammation, irritation, and infection [7,8,9,10,11].

The digestive system has evolved a unique mucosal immune system, maintaining a strong presence at the mucosal boundary of the GI tract, the gut-associated lymphoid tissues (GALT), consisting of lymphocytes, macrophages, and other immune-responsive cells [12]. This mucosal lining of the GI tract is the first immune defence, maintaining gut homeostasis and protection against pathogens [13]. Continuous surveillance and regulation through immune mechanisms that prevent chronic inflammation and restore homeostasis is critical in reducing a carcinogenic-promoting environment [14].

Inflammatory mediators disrupt normal homeostasis, antagonising and dominating homeostatic signalling to orchestrate a protective response against life-threatening infections and injury [15]. Acute inflammatory processes temporarily disengage and override homeostatic set point controls, and in states of acute and/or chronic inflammation, inflammatory cytokines have the capacity to dysregulate and reset these set point controls to an altered normal homeostatic base [16]. The unintentional disruption of the homeostatic reset control in the inflammatory process can potentiate long-term chronic inflammatory states that fail to resolve and underlie the well-documented association between inflammation and diseases such as diabetes and cancer [10,11,17,18]. Identifying and targeting mechanisms involved in, and responsible for, this altered stable state of homeostasis could be useful in the prevention, reversal, and treatment of inflammatory-based diseases. Bioactive sphingolipids, which include sphingomyelin, sphingosine (Sph), ceramide (Cer), sphingosine-1-phosphate (S1P), and ceramide-1-phosphate (C1P), derived from sphingolipids in the diet play an integral role in healthy GI immunity [19]. They are also involved in the regulation of inflammation associated with the pathogenesis of GI cancers. For example, ceramide affects T-cell immune signalling and can reduce tumour cell viability, supporting ceramide derivatives as potential candidates for tumour treatments [20,21]. A ceramide derivative, C6-ceramide, was shown to reduce tumour associated macrophages and suppress the anti-tumour suppressor response in a liver tumour model [20]. More recently, C6-ceramide was reported to reduce the cell viability of cutaneous T-cell lymphomas [21]. In this review, we focus mainly on another important bioactive sphingolipid, S1P, its role in immune homeostasis and its involvement in homeostatic dysregulation of the GI immune system, GALT, in inflammation [22,23,24,25,26,27,28,29]. S1P action in acute and persistent low-grade inflammation of the digestive system, from top to bottom, is discussed, with reference to a recent review by Sukocheva et al. [22]. We also provide an update on S1P modulators in clinical trials and in clinical therapy for GI inflammation, which may have longer term benefits in reducing the risk of GI cancers.

## 2. The Gastrointestinal Tract and Associated GI Organs

The GI tract is a continuous hollow tube constituting distinct organs linking the mouth to the anus with the primary function of absorbing nutrients and excreting waste [30]. The main GI tract is divided into the upper GI tract consisting of the mouth, oesophagus, stomach, and small intestine, and the lower GI consisting of the bowel, made up of the large intestine colon and rectum (colorectal), and the anus [31]. Major associated-digestive organs that feed into the major GI tract including the liver and intrahepatic bile ducts, the gall bladder and extrahepatic ducts, and the pancreas, are all at risk of inflammatory-associated cancers.

This continuous muscular tube harbours one of the largest luminal interaction areas and is lined with multiple mucosal epithelia, which have the fastest cell turnover within the body, consistently being shed and renewed on a weekly cycle through cell division, maturation, and migration [32,33]. The mucosal epithelia barrier plays a key role in the regulation of the immune system, maintaining constant immune-sensing, allowing absorption of nutrients, whilst limiting potential harm from antigens and pathogens [34]. Compromise or defects in the function of this GI mucosal barrier can occur in any part of the GI tract, resulting in various underlying aetiologies. Breakdown in this barrier affects the host–microbe balance, challenging the immune system and initiating an inflammatory reaction in the GI tract that is associated with diseases such as celiac disease, IBDs, and colon cancer [35]. GI-barrier immune defects also have consequences for extra-intestinal diseases including diabetes, obesity and chronic liver disease [35].

Although most strategies for treating GI cancer patients focus on the epithelial cell origin and epithelial compartmentalisation, in vivo evidence purports a more inclusive approach where epithelial cells respond to their microenvironment or matrix network of stromal, immune, and epithelial cells, and the enteric nervous system that controls gastrointestinal behaviour [36]. External stimuli, such as adverse environmental factors (high fat diets, toxins, and pathogens) can aggravate the digestive tract and induce inflammation.

Prime examples of digestive tumours arising under conditions of chronic inflammation, also referred to as persistent low grade inflammation, are oesophageal adenocarcinoma from repeated exposure to stomach acid (Barrett’s oesophagus), gastric cancers from exposure to Helicobacter pylori (*H. pylori*), colon cancer from IBDs, liver cancer from viral hepatitis, and pancreatic cancer from chronic pancreatitis [37]. Although there is a robust immune system in place to regulate the homeostatic state, chronic low-grade inflammation is notoriously problematic and challenging in this process. Prevention of this chronic immune response using anti-inflammatories was shown to reduce tumorigenesis [25]. However, timing is crucial in this process, balancing the necessary protective level of immune response to invading pathogens without the harmful side effects of chronic inflammation. Sphingolipids regulate normal physiological cellular processes, and play universal adaptive roles in immunity and inflammation in disease control [38].

Specific S1P receptor inhibitors have been developed, and are in development, to help elucidate critical steps in immunological processes that underlie many inflammatory-based diseases [39].

## 3. Sphingosine Kinase/S1P/S1P Receptor Pathways

**Figure 1:**
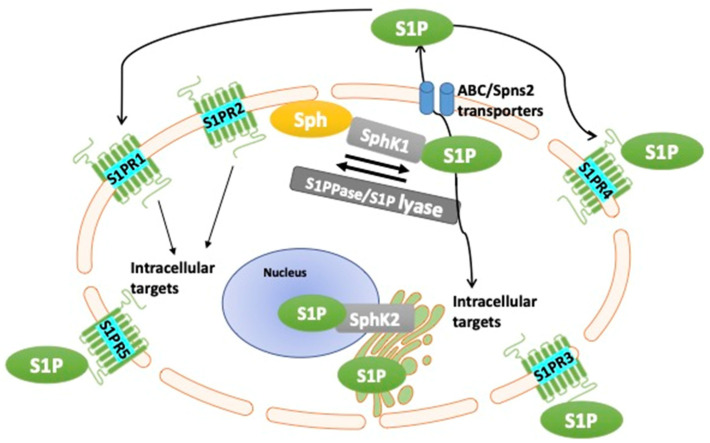
Sphingosine kinase (SphK)/sphingosine-1-phosphate (S1P) inside–outside model. SphK1 is primarily located in the cytosol and SphK2 is mainly localised in the inner mitochondrial membrane, the endoplasmic reticulum, and the nucleus. Sphingosine (Sph) is converted to S1P via SphKs and this process can be reversed via S1P phosphatases (S1PPase), which dephosphorylate S1P or are hydrolysed by S1P lyase to maintain dynamic equilibrium. S1P catalysed by SphK1 is transported outside the cell via ABC/Spns2 transporters where S1P binds to S1P transmembrane receptors (S1PR1-5). Binding of S1P to its cognate receptors on self and other cells activates intracellular signalling pathways. The SphK2/S1P pathway regulates processes in the nucleus such as transcription and telomere maintenance, as well as processes in the mitochondria, and is involved in mitochondrial respiration [52].

Sphingolipids have diverse multi-functional activities in homeostasis of the body, ranging from their traditional role as integral membrane structures, to their more intriguing role as mediators of cell death and survival, adhesion, migration, intracellular trafficking, calcium regulation, angiogenesis, and in the mediation of immune cell function and inflammation [8,23,40,41]. The S1P molecule (2S-amino-1-(dihydrogen phosphate)-4E-octadecene-1,3*R*-diol) is a membrane-derived lysophospholipid [42]. The derivation of S1P is mainly through the metabolism of sphingomyelin at the plasma membrane, which is enzymatically converted to ceramide, ceramide is deacylated by ceramidases to yield sphingosine, and sphingosine is further phosphorylated to its active form, S1P, by sphingosine kinases (SphK1 and SphK2 isoenzymes and their isoforms) [43,44,45,46,47]. Activation of SphKs catalyses the increase in S1P levels whereby S1P can function as an intracellular second messenger or can be transported outside the cell via ABC/Spns2 transporters acting in an autocrine and/or paracrine manner to activate S1P receptors on the cell surface [48]. This mechanism of action is known as the inside–outside signalling of S1P (Figure 1) The levels of S1P inside the cell are tightly regulated by SphKs and S1P phosphatases (S1PPase) or S1P lyases [48]. The two SphK isozymes (SphK1 and SphK2) are located in different sub-compartments of the cell; SphK1 is localised in the cytosol, whereas SphK2 is localised in the nucleus, the inner mitochondrial membrane, and the endoplasmic reticulum [46,47]. The SphK1 isoenzyme is also released into the extracellular fluids, acting to directly phosphorylate sphingosine to its active form (S1P) in this extracellular environment [49]. Based on SphK1-null and SphK2-null mice experiments, there is some indications to suggest intracellular SphK1 is proinflammatory and SphK2 plays an anti-inflammatory role, with the reservation that SphK1 and SphK2 have significant redundancy in their functions [50,51].

## 4. SIP/S1PR Inflammatory Response

### 4.1. S1PR1-5 Localisation and Functions

**Figure 2:**
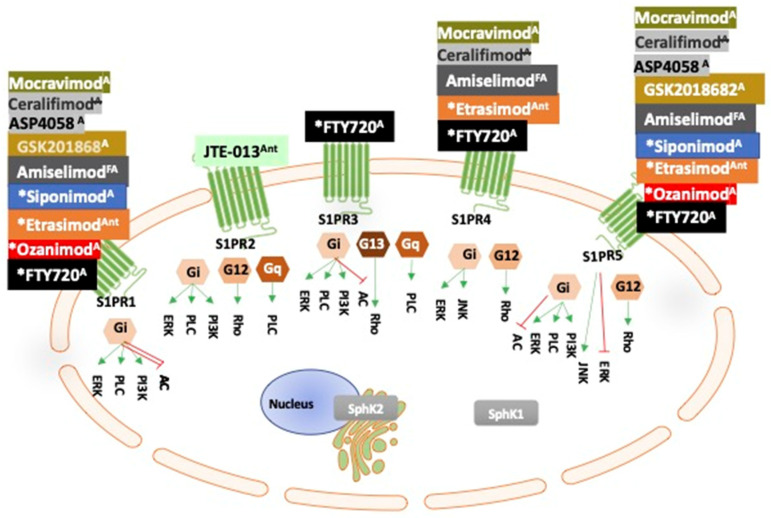
Sphingosine-1-phosphate receptor (S1PR) modulators in clinical trials for gastrointestinal cancers (GI) (adapted from [47]). Extracellular S1P binds to S1P transmembrane receptors (S1PR1-5) coupled to different G-proteins, which activate different internal signalling pathways within the cell. S1PR modulators bind to one or more of the S1PRs to block or activate the S1PR signalling as illustrated. Each of the S1PR modulators, as illustrated, are currently registered with National Institute of Health (NIH) clinical trials to determine their efficacy in GI inflammatory disease. * Denotes S1PR modulators approved for clinical use (see Table 3). JTE013 is a competitive antagonist specific for S1PR2 (only in pre-clinical use). ^A^ denotes agonist; ^Ant^ denotes antagonist. ^FA^ denotes functional antagonist. Note: S1PRs effects are dependent on timing and concentration of the modulator.

Inflammation is the host’s immune defence against potential or actual harm by environmental insults whereby immune cells are recruited to the site of damage for repair [53]. If repair is not effective and damage continues, the conundrum is, the persistent invasion of inflammatory cells to the damaged site causes chronic inflammation eventually undermining the body’s resistance to inflammatory diseases such as cancer [11,16,54,55]. The maintenance of relatively high concentrations of S1P in the extracellular fluids commands a critical role for S1P in maintaining homeostasis and, importantly, in the control of inflammatory responses including cancer and diabetes, and, more recently, it was suggested that S1P may play a role in chronic COVID-19 inflammation [8,29,39,56,57]. Extracellular S1P binds and signals through S1PRs, also known as EDG isoforms, which belong to the G protein-coupled transmembrane receptors on the cell surface [58,59] (Figure 1). However, it is also noted that S1P activity can occur independent from binding to the S1PRs [48]. SIP binding to S1PRs is a critical step in cell trafficking, blood vessel development, and maintaining homeostasis [60]. There are five S1PRs (S1P1-5) each with different nanomolar dissociation constants, which are important for different signalling events and cell functions in physiological and pathophysiological processes [61]. However, individual S1PRs are not equal in their importance in homeostasis and their potential roles in pathogenicity [62,63]. For example, S1PR1 is the most studied and is essential for embryological development including the formation of the vasculature; S1PR1-null mice do not survive [64]. Additionally, not all S1PRs are expressed on all cells, and individual S1PRs are coupled to distinct intracellular G-alpha subunits (Figure 2), which contribute the diversity of S1P signalling in normal development processes in the body and are also contributory factors in pathogenicity [60]. The S1P receptors 1–3 are principally expressed in the vascular endothelium, the immune system, and in the central nervous system, S1PR4 is mainly expressed in the lymphoid tissue, and S1PR5 is predominantly found in the immune natural killer cells, the spleen, and the central nervous system [39,65,66]. S1PR1 is critical in the regulation of inflammatory processes driving neovascularisation, providing tumours with the nutrients and oxygen needed for cancer cell survival, with S1PR2 and S1PR3 having some compensatory functions in the absence of S1PR1 [57].

The different S1P receptors play distinct or overlapping roles in the innate immune response, in the trafficking, differentiation, and activation of immune cell effector functions [67,68]. S1PRs1-5 are differentially expressed on different innate immune cell subtypes, summarised in Table 2 alongside the proposed innate immune functions of the binding of S1P to each of the S1P receptors. S1PR1 is the most well studied and ubiquitously expressed on all immune cells, both S1PR2 and S1PR4 are expressed on macrophages, monocytes, eosinophils, and mast cells, S1PR3 is expressed on neutrophils during inflammation as well dendritic cells, and S1PR5 is expressed on patrolling monocytes and natural killer cells.

During inflammation, proinflammatory cytokines stimulate the production of S1P through SphK action [69]. Extracellular S1P activation of S1PRs on the different cell types is particularly important in driving the diverse immune cell inflammatory response [45]. S1PR1’s ubiquitous expression on immune cells is associated with the stimulation of anti-inflammatory responses and apoptosis in macrophages to trafficking of the dendritic and monocyte cells, the inhibition of IFN-α secretion, and the recruitment of eosinophils and mast cells, and is important in lymph node egress of the natural killer cells [67,68]. The functions of the S1PR2 complex include eosinophil and mast cell degranulation [70]. The S1PR3 complex is involved in the suppression of regulatory T-cells, promotion of Th1 response, and maturation of the dendritic cells, and is also involved in the trafficking of the monocytes and recruitment of the mast cells and eosinophils [67,71]. In dendritic cells, the S1PR4 complex is associated with plasmacytoid differentiation and the inhibition of IFN-a secretion, and the S1PR4-complex is also associated with the recruitment of mast cells, eosinophils, and monocytes [67,72]. S1PR5 has limited expression in the immune cells; however, its expression on natural killer cells is associated with bone marrow egress, and on monocytes it has a role in monocyte trafficking [73].

### 4.2. Maintenance and Function of S1P in Blood and Lymph Vessels in Inflammatory Response

Plasma-S1P is a key lipid mediator in the innate and adaptive inflammatory immune cell response in the GI tract [68,74]. Conserving a readily available rich source of S1P is essential for S1P-S1PR innate immune function, such as the recruitment of inflammatory cells to the site of damage, in addition to preserving the integrity of the blood vessels [41]. Freestanding S1P has a rapid turnover of approximately 15 min and intracellular SphKs (SphK1 and to a lesser extent SphK2) constitutively contribute to the extracellular egress of S1P to maintain S1P levels [68,75]. The main sources of circulating plasma-S1P production are the red blood cells, platelets, fibroblasts, and vascular endothelial cells [68,74]. S1P concentrations are naturally high in the blood and lymph for essential blood and lymphatic vessels’ functions, especially in the regulation of the inflammatory response [76]. S1Ps carried in the blood stream by albumin (albumin-bound-S1P) and the high-density lipoprotein (HDL) apolipoprotein M (ApoM) binds to and activates S1PRs on neighbouring or distant cells [77,78,79,80]. S1P’s physiological function appears to be dependent on whether it is bound to albumin or HDL-ApoM; HDL-ApoM binds to S1P in a more stable complex and is key in vascular and endothelial preservation [77]. HDL-ApoM preferentially targets cells expressing S1PRs 1–3 and induces S1PR internalisation and Gi signalling pathways [77]. More recently, HDL-ApoA4 was identified as a S1P carrier [81]. Animal experiments suggest that the S1P-HDL-ApoM complex is essential for the stability of S1P in embryological development; however, albumin-bound-S1P can compensate, in part, for ApoM function [78]. Recently, S1P complexing with ApoA4 albumin-bound-S1P was shown to compensate for S1P-HDL-ApoM activity [81].

In acute and chronic inflammation, plasma-S1P has been shown to be protective, as demonstrated in animal models [41,82]. In response to acute inflammation, increased binding of plasma-HDL-ApoM-S1P to S1PR1 was shown to maintain vascular barrier integrity through inducing adherent junctions, suppression of VEGF, and proinflammatory cytokine signalling [41].

In the lymphatic system, S1P signalling also plays a key role in the immune response to inflammation [83,84]. The S1P concentrations are strictly maintained as a gradient between the systemic circulation and lymphoid tissues to facilitate lymphocyte trafficking [68]. S1P is degraded by S1P lyase in the lymphoid tissues to maintain this gradient; this occurs between the blood/lymph (high S1P) and lymphoid tissues (low S1P) [85]. This gradient allows newly formed T-cells to egress from the thymus into the circulatory fluids and the traffic of mature T-cells and B-cells from the secondary lymphatic organs [83,84]. Both S1PR1 and S1PR5 are key in lymph node egress. The S1P/S1PR5 complex was found to be necessary for natural killer T-cell trafficking from the lymph nodes and bone marrow [68].

### 4.3. S1P Intracellular Signalling and Inflammatory Response

Attribution of S1P inflammatory action and immune regulation is mainly as a first messenger by extracellular S1P binding to the S1PR1-5 transmembrane proteins on different immune cell types, as discussed. S1P also acts as an intracellular second messenger by direct stimulation of intracellular signalling proteins involved in inflammation and pro-survival. Intracellular S1P has been reported to contribute to the inflammatory response via several different pathways. Inflammatory cytokines such as tumour necrosis factor (TNF-α) are secreted by the inflammatory cells as part of the antigenic immune response. TNF-α was shown to activate the SphK1/S1P pathway and is proactive in endothelial cell activation and adhesion molecule expression [86]. TNF-receptor-associated factor 2 (TRAF) is part of the TNF multi-component signalling which binds to SphK1 and is important in mediating TNFα-stimulated nuclear factor kappa B (NFkB) to induce proinflammatory mediators and cell survival [87,88]. Cytoplasmic-generated S1P can also function as an epigenetic co-regulator in lipopolysaccharide (LPS)-induced interleukin 6 (IL6) and can also stimulate reactive oxygen species (ROS) [89]. Within the nucleus, S1P, generated by SphK2, can inhibit histone deacetylases 1 and 2 (HDACs1/2) to alter histone acetylation and initiate inflammatory-responsive gene expression [52,90].

## 5. Impact of Dietary Sphingolipids in GI Inflammation

### 5.1. Saturated Fatty Acids (SFA) and Inflammation

Disruption of the sphingolipid metabolism through the oversupply of saturated fatty acids (SFA) in a HFD is associated with low-grade systemic inflammatory processes. HFD increases the amount of sphingolipid metabolites affecting the downstream sphingolipid-mediated cellular signalling pathways [91,92]. Dysregulation of sphingolipid homeostasis derived from an unhealthy nutritional fat-rich diet is associated with increased risk of immune-related inflammatory diseases such as diabetes and cancer [29,92]. Dietary sphingolipids are not only ubiquitous key structural building components of our cell membranes, but they are also the centre of many biological signalling processes that are essential for homeostasis [93]. The imbalance of sphingolipids, ceramide, sphingosine, and S1P, in both pro- and anti- inflammatory responses, and their role in inflammatory diseases within the cellular environment are well documented [23,27,38,41,43,91]. Direct dietary sphingolipid action within the gut is less well studied but is emerging as an interesting phenomenon in the changing gut biome [19].

### 5.2. Direct Effects of Dietary Sphingolipid Metabolites and the Gut Biome

Digestive disorders are linked to an imbalance of the gut microbiome resulting in low-grade gut inflammation [13]. The dietary source of sphingolipids, as discussed by Norris et al. [94], is potentially an important factor in inflammatory diseases. These dietary sphingolipid metabolites have been shown to directly influence gut immune homeostasis both positively and negatively, through inducing changes in the gut microbiota [94]. A positive example, sphingolipid metabolites have been shown to be protective by competing with commensal bacteria for intestinal attachment to help in the prevention of pathogenic invasion [19]. Alternatively, excess sphingolipids from HFDs can negatively influence gut homeostasis; they have the potential to promote inflammation by negatively altering the gut microbiome and disrupting GI barrier function by inhibiting intestinal lipid absorption [94]. Imbalances in sphingolipid signalling influence the mucosal–bacterial interaction’s involvement and play a role in chronic low-grade inflammation [19]. Potential effects of dietary sphingolipids in the acute and chronic inflammatory response include maintenance of gut health (microbiome and intestinal inflammation), lipid metabolism (fat and cholesterol absorption), and endotoxemia (dissipation of lipopolysaccharides (LPS) and prevention of LPS translocation) [94]. Direct anti- or pro- inflammatory effects of consuming dietary sphingolipids in the gut were shown to be distinct from the effects of cellular sphingolipid inflammatory signalling, as reviewed in [19].

Gaining recognition as immune influencers of gut inflammation are gut bacteria-produced sphingolipids. Sphingolipid metabolites produced by some microorganisms within the gut biome were shown to directly impact the host metabolic pathways and host immune response and GI barrier function [95]. For instance, the Bacteroidetes phylum, which is dominant in the gut, and some opportunistic pathogens produce sphingolipid metabolites that are structurally similar and can act comparably to human sphingolipids [96]. These bacterial sphingolipid metabolites (such as ceramide and S1P) can also mediate specific immune responses. For example, bioactive S1Ps are mainly derived from ingested dietary sphingomyelin converted intracellularly by SphKs [19]. Sphingolipid-producing bacteria have been shown to produce a S1P-like metabolite that is recognised by the S1P receptors (or G- protein-coupled receptors (GPCRs)). These S1P-like metabolites have the potential to bind to the S1PRs located on the surface of the intestinal endothelial cells and effect S1P-signalling and S1P-mediated host responses [96]. Further investigation into the nature and contribution of dietary sphingolipids in the gut-will help in the understanding of the role sphingolipids in chronic, low-grade inflammation and disease prevention and treatment.

## 6. Role of S1P/S1PR in Head and Neck (Mouth/Throat/Salivary Glands) Cancers

Head and neck cancers are heterogenous tumours arising from the lips, oral and nasal cavity, sinuses, salivary glands, and pharyngeal compartments and larynx, mainly comprising squamous cell carcinomas [97]. Head and neck squamous cell carcinomas (HNSCCs) are aggressive, and express various cytokines and growth factors, making them very inflammatory in nature [98]. Resistance to chemotherapy, radiation therapy, and targeted therapies is common and late-stage detection survival rates are low.

The presence of intracellular elevated SphK1 expression correlates with clinical failure and poor survival in HNSCC patients and S1P-targeted therapy was put forward as a possible inclusion for resistant, hard-to-treat, HNSCC tumours [97]. A role for SphK1/S1P was first demonstrated by Shirai et al. in 2011 where they showed SphK1 over-expression was characteristic in all HNSCC tumours they tested (stages I–IV), [99]. In their mouse model, SphK1^−/−^/KO, HNSCC was significantly reduced, and they speculated that SphK1 activation was required to induce proinflammatory cytokines (including the interleukins IL-1b and IL-6) in the mediation of inflammation-related HNSCC cancer development [100]. They also demonstrated this in colon colitis and carcinogenesis [100]. Later in vitro studies supported SphK1 modulation of proinflammation in HNSCC cell lines through S1P/interleukin-6 (IL6)- and S1P1/ERK STAT3 signalling [101]. Elevated SphK1 leads to an S1PR1/ERK- and IL-6 /gp130- mediated increase in proliferation, migration, and inflammatory and a more aggressive HNSCC phenotype [101]. Indirect targeting of the sphingolipid pathway was demonstrated to sensitise chemo- and radiotherapy-resistant HNSCC, supporting their clinical usefulness in hard-to-treat tumours [102]. Indirect inhibition of SphK1 using a targeted microRNA, miR-124, was shown to suppress HNSCC [103].

Oral squamous cell carcinoma (OSCC) is the most common cancer of the HNSCCs [104]. There is increasing emphasis on nutrition associated with changing host oral microbiome linking bacterial inflammation, immunosuppression, abnormal energy metabolism, and carcinogenesis [104]. In OSCC patients, S1P-metabolising enzymes, after measuring the mRNA levels, were found to be significantly altered, correlating with clinicopathological attributes and host metabolism, with the expression of SphK1- and S1P-metabolising enzymes found to be significantly upregulated in OSCC patient tumours [105].

Currently, there is increasing emphasis on the effects of oral microbiota dysbiosis, which not only increases inflammatory OSCC, but several periodontal disease-associated species were discovered to increase GI tract cancer risk [106]. Systemic disturbances in the microbiome throughout the digestive system, whether through nutrition or pathogens, are a continuous underlying theme that links increasing cancer risk of the GI tract and associated organs.

Salivary gland cancers are a rare heterogeneous family of tumours with a little-known aetiology. Few studies have explored the role of sphingolipids in salivary glands, even though, in human salivary gland cancers, SphK1 expression was found to be significantly correlated with clinical stage and poor survival [107]. Hence the suggestion that SphK1 can be used as a biomarker and potentially as an adjunct therapeutic target for salivary cancers. Studies of sphingolipid metabolism in salivary glands in rats fed on a HFD demonstrated a change in the sphingolipid composition, with significant accumulation of S1P in the salivary glands of rats resulting from chronic high-fat feeding [108]. Although these rat studies were focused on obesity and diabetic effects, they do support the association of S1P in inflammatory salivary gland diseases.

Direct clinical evidence to support sphingolipid-based therapies for HNSCC, including salivary gland cancers, however, is still very limited and is mainly based on inference from other S1P-therapy-based cancer studies and clinical trials in solid cancers [102].

## 7. Overactive S1P/S1PR Promotes Oesophageal Cancer, Invasion, and Metastasis

One of the deadliest cancers worldwide is oesophageal cancer, with a poor prognosis and high mortality rate (Table 1). Oesophageal cancers mainly (>90%) consist of adenocarcinomas (cancer formed from the glandular structures in the epithelial tissue) and squamous cell carcinomas (cancer cells in the epidermal layer of the oesophagus) [109]. A highly inflammatory oesophageal environment caused by gastroesophageal reflux diseases (GERDs) and Barrett’s oesophagus are well-known pre-malignant conditions for oesophageal adenocarcinomas [110,111]. Oesophageal cancer cells’ main route of migration is through the lymphatic system [112]. Lymph node metastasis and aggressive invasion of neighbouring organs occurring early in disease onset makes these cancers particularly notorious to effectively treat and, therefore, patients have a poor prognosis.

The role of SphK/S1P in oesophageal cancers has been extensively reviewed [22]. Key points to note are the differential and increased expression of SphK and S1PRs during the mesenchymal transition and the aggressive invasive stages of oesophageal squamous cell carcinoma; increased levels of SphK1 and serum S1P all correlated with metastasis-positivity in lymph nodes in most oesophageal cancer patients [113]. In 2011, Pan et al. [114] identified SphK1 as a key mediator of aggressive, invasive, oesophageal cancer cells and suggested that blocking this pathway may be a way forward to disruption of the metastatic phenotype and more efficacious treatment. In in vitro models, Pan et al. demonstrated that invasion and metastasis of oesophageal cancers correlated with enhanced phosphorylation of epidermal growth factor receptor (EGFR), upregulation of SphK1, and upregulation of EREG (epiregulin) and AREG (amphiregulin), which preceded tumour invasion [114]. To compliment these in vitro studies, the association between oesophageal cancer lymphatic migration and high SphK1, correlating with poor patient clinical outcome, was reported in a study by Nemoto et al., 2019 [115].

Differential expression and localisation of S1PRs have been shown to influence oesophageal cell metastasis. S1PR5 overexpression in mucosal oesophageal cancer cells, in vitro, is associated with a decreased S1P-induced proliferation and migration [116]. Alternatively, high expression of S1PR2 on mesenchymal-like oesophageal cancer cells is associated with the invasive phenotype in this highly acidic gastroesophageal reflux environment [117]. Hence the suggestion that targeting S1PR2 with a specific S1PR2 antagonist, JTE-013, may be a potential adjunct therapy for these types of oesophageal cancers.

## 8. SphK/S1P/SIPRs Contribution to Gastritis and Gastric Cancers

Gastric or stomach cancers can be triggered by inflammation of the gastric epithelium caused by constant onslaught of opportunistic pathogenic infections, including the bacteria *H. pylori* and the Epstein–Barr virus [118,119,120,121]. Whilst most viruses and bacteria are eliminated by activated immune cells, tumorigenic pathogens can evade the host immune-defence, enabling persistent chronic infection and inflammation [121]. For example, *H. pylori* survives the acidic gastric environment and irritates and destroys the protective mucosal lining of the stomach, making the epithelial cells lining the gut more vulnerable to carcinogenesis. Changes in the stomach lining can lead to severe inflammation, ulceration, and chronic gastritis, characterised by enhanced inflammatory gene expression [122]. The understanding is that persistent infection indirectly induces cancer through the recruitment of immune cells, which produce high levels of proinflammatory cytokines, induce oxidative stress, and are precursors of malignancy in the stomach [123]. As discussed, gut microbiota dysbiosis, defined as a reduction in microbial diversity of the GI tract and a loss of beneficial bacteria, and/or, in some cases, an increase in opportunistic pathogens, also impedes mucosal barrier function, leading to chronic low-grade inflammation of the gut [19]. In animal models, HFDs have been shown to promote inflammation in the intestines and alter the permeability of the gut [94,124,125]. Sphingolipids have been shown to play important roles in influencing pathogenicity directly in the gut microbiome and indirectly through regulation of the innate, adaptive, and chronic hyperinflammatory immune response [19,40]. A comparison of gastric cancer and normal tissue specimens showed that gastric cancer tissue generally had reduced sphingosine-1-phosphate phosphatase 1 (SGPP1) [126]. SGPP1 dephosphorylates S1P into sphingosine and tips the balance of S1P to sphingosine. In vitro experiments showed that knockdown of SGPP1 increased invasiveness and migration of gastric cancer cells [126]. SphK1 was also shown to be elevated in gastric cancer tissues, correlating with poor prognosis [127,128].

Screening and profiling of gastric cancer cells by Yamashita et al. [129] revealed differential expression of S1PR receptors; all gastric cancer cells expressed S1PR2, with a noted absence of S1PR4 and S1PR5. Variable S1PR1 and S1PR3 expression was observed in some, not all, gastric cell lines tested. This observation was significant given the opposing action of S1PR2 and S1PR1/S1PR3; S1PR2 inhibits tumour migration whereas S1PR1 and S1PR3 promote migration. In vitro experiments by Li et al. [128] demonstrated a correlation between elevated S1PR1 and gastric cancer cells’ migration. In animal models, Zhou et al. [127] demonstrated that S1P-S1PR1 signalling promotes gastric cancer progression by increasing the expression of cytokines and recruitment of myeloid-derived suppressor cells to the tumour microenvironment, thus impairing the anti-tumour functions of the tumour-infiltrating lymphocytes (TILs). In this animal model, a specific S1PR1 agonist, SEW-2871, which activates S1PR1, blocked cytotoxic T lymphocyte anti-tumour function [127]. S1P is linked to persistent STAT3 activation, chronic intestinal inflammation, and colitis-associated cancer [130]. In tissue array studies, enhanced S1PR1 co-localising with STAT3 was commonly seen in higher-grade GC tumours; GC patients with high S1PR1-STAT3 expression responded poorly to chemotherapy drugs and blocking S1PR1-STAT3 signalling re-sensitised drug resistance in GC cells [131].

These observations and experiments, as well as many others, provide insights into the contribution of sphingolipids in gastric cancer development, providing important information for potential early therapeutic anti-SphK and anti-S1PR intervention targets in the prevention of metastasis in gastric cancers.

## 9. S1P and Small Intestine/Colorectal/Anal Cancers

The lower intestine, colon, rectum, and anus are all encompassed in the category of bowel or colorectal cancers. Colorectal cancers are immune-mediated diseases that are collectively referred to as IBDs and characterised by a lifelong, relapsing inflammation that can occur throughout the intestinal tract through to the anus. Most studies of colitis-associated cancer (CAC) exemplify the link between inflammation and the pathogenesis of cancer; the incidence of developing CAC rises considerably in patients affected by IBDs, with the two well-defined major sub-groups, CD and UC [132,133,134]. The first observation between chronic inflammation underlying the incidence of CD can be credited to the German surgeon Wilhelm Fabry in 1623; however, centuries later, in 1925, this disease was named after the American physicist, Burril B. Crohn, who made ground-breaking advances in the identification of this disease [135].

Similarly to gastric cancers, colorectal cancers have a common grounding, microbiota dysbiosis, typified by a reduction in microbiome diversity and a disruption in the mucosal and epithelium lining, as well as persistent, chronic low-grade inflammation [136]. The mucosal immune system within the gastrointestinal mucosa forms a semi-protective barrier against pathogens, and whilst many inflammatory mucosal responses are self-limiting, an abnormal mucosal immune response, in contrast, is thought to result in chronic inflammation resulting in IBDs [137].

Disruption of the microbiota in the intestinal tract can be due to an imbalance of good and bad bacteria (a reduction in microbial diversity and beneficial bacteria), viruses, fungal inhabitation, medication [138], or simply bad eating habits [136].

SphK1, S1P, and S1PRs are gaining increasing importance as generic regulators of inflammatory immune responses, as discussed above, and the importance of the role of cellular SphK-S1P-S1PR action in colon cancers has been covered in a couple of recent reviews and book chapters [22,40]. There is a consensual agreement that SphK1 and S1P expression is elevated in UC and colon-associated cancers [38]. In human colon cancer progression and metastasis, SphK1 and S1P were shown to have higher expression compared to those without cancer/metastasis [100]. In vivo, in a murine colon cancer model, SphK1 and S1P were found to be significantly elevated in the mucosa compared to normal mucosa, with a concomitant increase in S1P levels in mice with colon cancer [100].

In addition, in the colon, SphK1 regulates the inflammatory cyclooxygenase-2 (COX-2) [139], and elevated COX-2 is both a biomarker for poor patient outcome and a therapeutic target for colon cancers [140,141]. In a SphK1-knockout model, mice showed partial resistance to chemically induced colitis, and in cancer-associated colitis, significant attenuation of colon cancer was achieved [100]. Recently, it was suggested that SFA in the diet directly stimulates SphK1 inflammatory responses (COX2, TNFα, JNK) bypassing the need for S1P and S1PR activation, as demonstrated in intestinal epithelial cells [142]. In vitro studies also support the promotion of the epithelial–mesenchymal transition in colon cancers by SphK1 mediating the focal adhesion, protein kinase B (AKT), and matrix metalloproteinase (MMP)2/9 pathway [143].

Rectal cancers comprise approximately 25% of colorectal cancers, sharing many similar features to colon cancers [144]. A distinct difference between the proximal colon and the rectum is the rectum does not have the same protective serosa outer layer found in the colon; hence, tumours are more likely to be invasive and have a much higher prediction of recurrence [145,146]. Rectal cancers can have devastating effects on fundamental bodily functions such as bowel movements, urination, and sex. Rectal cancers have significant intra-tumour genetic heterogeneity, making them difficult to treat [147]. Chronic inflammation is a major risk factor for rectal and anal cancers; patients with chronic Crohn’s disease or chronic ulcerations are at high risker of rectal cancers and/or anal cancers in the fistula-lining epithelium [148,149]. The etiologically of cancer of the most distal region of the rectum, the anal canal is also associated with chronic inflammation caused by viruses, human papillomavirus (HPV) infection, and human immunodeficiency virus (HIV) [150].

Biologics (biological agents targeting specific inflammatory pathways), which include immunomodulators and anti-tumour necrosis factor (anti-TNF), are at the forefront of the pharmaceutical management of IBDs [151]. The lack of primary and acquired response to anti-TNF therapy in 20–40% of patients has necessitated the development, approval, and application of new anti-inflammatory treatment options, reviewed in [151].

Preclinical trials have demonstrated the benefits of using S1PR1 modulators in IBD therapy to modulate intestinal leukocyte migration by reducing inflammatory immune cells into the mucosa, reducing inflammatory cytokines, and expanding regulatory T-cells [152]. The S1PR immunomodulator, Fingolimod (2-amino-2[2-(4-octylphenyl)ethyl]-1,3-propanediol), FTY720, is believed to act by facilitating the internalisation of S1PR1 on lymphocytes, thus inhibiting the migration of lymphocytes in the S1P gradient from the lymph nodes to the site of inflammation. Thus, the lymphocytes are retained in the lymph nodes and repression of the inflammatory response occurs.

S1P/S1PR modulators are now at the forefront in the arsenal of new treatments under development for IBD, with several second-generation S1PR1 modulators currently in clinical trial to treat UC (Table 3). The recent FDA approval of ozanimod (Zeposia^TM^, Bristol Myers Squibb, New York City NY, USA) for adults with moderately to severely active UC is the first S1P receptor modulator recently approved for UC [153]. Treatment with Zeposia^TM^ for UC has the potential to also reduce colorectal cancers, with UC being a major precursor for colorectal cancers.

## 10. Liver Cancers, Inflammation and SphK/S1P

### 10.1. Liver Cancers

There are two major types of primary liver cancer: hepatocellular carcinoma (HCC), which arise from the hepatocytes and represent 80–90% of cases, and intrahepatic cholangiocarcinoma, a group of bile duct cancers comprising around 10–15% of cases. The liver is home to many secondary metastatic cancers and these resident metastatic cancers are 18–40 times more common than primary liver cancers [157]. Approximately 50% of liver metastasis arise from the colorectal region, and the rest mainly from the GI cancers of the oesophagus, the gut, and the pancreas, as well as intestinal neuroendocrine tumours and GI stromal tumours. Primary liver cancers almost exclusively stem from an underlying chronic inflammation [158]. Chronic inflammation causes changes in liver lipid metabolism and increases cancer risk [153,159,160,161]. Lipid metabolism disorders create favourable microenvironments for tumour growth, with non-alcoholic fatty liver disease being identified as a major precursor for liver cancer [162]. Indisputably, chronic inflammation of the liver untreated will interfere with liver function, increase the risk of liver failure and end stage liver disease, and is a high-risk factor for primary cancers.

### 10.2. Obesity, S1P and Inflammation in the Liver

In the liver, SIP is essential for the maintenance of normal liver homeostasis and is emerging centre stage in liver pathobiology [163]. Obesity alters the sphingolipid signalling pathways and obesity was found to increase blood S1P levels in humans and mice [163]. Lipid overload from a high fat diet is increasingly seen as a significant contributing risk to diseases such as diabetes and cancer [164,165]. HFDs are characterised by high levels of palmitate, which are precursors of sphingolipids such as ceramide [163]. Fatty deposit accumulation in the liver is highly common in overweight and obese individuals. These individuals have a higher chance of chronic inflammation and liver fibrosis. Increased levels of free fatty acids and their metabolites resulting from excessive nutrition (HFD and high calorie intake) can induce lipotoxicity, cellular damage, hepatocyte necrosis, inflammation, steatohepatitis, and hepatic primary cancers [165]. For example, HCC, the most prevalent form of liver cancer, is frequently preceded by fatty lipid deposits and inflammatory-induced cirrhosis [162]. S1PR2, which is highly expressed in hepatic tissue and in the GI tract, and SphK2 are important regulators of hepatic lipid metabolism [166]. SphK2 inhibits HDAC activity and SphK2 knockout mice had reduced gene expression of some genes involved in hepatic metabolism [166] The role of S1PR2 was less clear but the suggestion was that mice lacking S1PR2 were not able to upregulate SphK2 in response to HFD [166].

The association between SphK1 and hepatic inflammation was also demonstrated in a mouse model of non-alcoholic steatohepatitis, where high saturated fat feeding initiated proinflammatory signalling in hepatocytes through the SphK1/S1P/S1PR1 pathway [167]. S1P was found to be elevated in obesity and correlates with metabolic abnormalities [168]. There is increasing evidence to support the hypothesis that metabolic perturbations result in a reduction in cellular ceramide levels and in an increase in SphK1 and S1P to promote HCC, with a negative outcome [162,169].

### 10.3. S1Ps Role in Liver Injury and Inflammation

Activation of S1PRs by S1P is also involved in the promotion and recruitment of bone marrow mesenchymal cells, which differentiate into hepatic stellate cells in the liver [19]. In liver injury, S1P promotes the stellate cells’ transdifferentiation into myofibroblasts, which secrete fibrotic components to form the extracellular matrix. S1P is an important mediator of fibrosis, inflammation-induced liver injury. In response to injury, S1P is released from the liver and aids in the recruitment of immune cells including Kupffer cells (liver-resident macrophages), which induces hepatic inflammation [19]. Importantly, the liver manufacturers and secretes apoM, which binds S1P and HDL to maintain endothelial barrier integrity. Hence, S1P plays a key role in maintaining hepatic homeostasis.

### 10.4. A Role for Apoprotein M (ApoM/)-S1P in Liver and Distal Cancers

The apoM-S1P complex was found to play a central role in numerous inflammatory and lipid metabolism disorders, including hepatic diseases (liver fibrosis, hepatic infections and sepsis, steatohepatitis, liver injury, and HCC) [170]. The liver is the main source of apoM, a major carrier of S1P in the blood, which is produced by the hepatocytes, and was shown to influence and enhance S1P biosynthesis [171,172]. ApoM, when bound to S1P, delivers S1P to extrahepatic tissues and plays a key role in immune functions, as discussed previously (in the “Maintenance and function of S1P in blood and lymph vessels in inflammatory response” section) and reviewed in [38]. Reduced levels of ApoM were found to impair liver function, and when a liver is damaged, ApoM levels decrease [173]. Deficiency of ApoM plays a critical factor in liver steatosis and, in vitro, a lack of ApoM promotes tumour cell survival by blocking liver cancer cells’ apoptosis [173]. Bai et al. [173] provide in vitro and in vivo evidence that producing and maintaining ApoM levels in the liver may be protective against liver cancer and metastasis, and overexpression of ApoM induces apoptosis. Deletion of ApoM in the mouse model was shown to increase migratory and invasive potential of mouse liver cells. Further support for this theory can be found—in an analysis of 50 matched primary liver tumours and adjacent matched normal tissue, ApoM was lower in the cancer liver tissue samples [173]. Damaged liver, by environmental insult, particularly poor nutrition, leads to disruption/dysfunction in lipid metabolism, including the deregulation of ApoM, thus providing a niche for liver carcinogenesis. Disruption of ApoM production in the liver also has longer-term holistic consequences for S1P transport and natural bodily function. Reduction in apoM’s availability to bind to circulating S1P causes endothelium dysfunction, chronic inflammation, and diseases ranging from cardiovascular diseases, cancer, and infections such as sepsis [174]. Understanding of the apoM-S1P axis/signalling is still in its infancy [170,173,174]. Compared to studies on S1P, the studies on the apoM-S1P axis are relatively few, but of interest due to its emerging importance in inflammatory diseases, in particular its role in hepatic diseases and HCC [170].

## 11. A Role for S1P in Biliary Tract Cancers

### 11.1. Biliary Tract Cancers

Biliary tract cancers (BTCs) are a rare, heterogeneous, highly metastatic, lethal group of cancers that include intrahepatic, perihilar and distal cholangiocarcinomas, and gallbladder cancers (GBCs) [175,176,177]. Although the gall bladder and bile duct cancers are both part of the same drainage system, they are two separate cancers. Due to the low occurrence of biliary and gall bladder cancers, they are usually studied jointly although GBCs are clinically and molecularly distinct and respond differently to radio- and chemotherapy [178].

The risk of biliary cancer includes inflammation of the bile duct (primary sclerosing cholangitis) and has a strong association with IBDs, obesity, and diabetes. An extensive meta-analysis by Li et al. [179] links overweight and obesity to significant increases in the risk of GBCs and extrahepatic bile duct cancers. Historically, the major role of bile is to break down fats into fatty acids and it is not surprising that being overweight and/or obese are linked to altered bile acid metabolism and increased risk of GBCs and extrahepatic bile duct cancers [179]. In the last decade, studies have revealed that bile acids are pleotropic and activate major complex signalling events including mucosal immunity and inflammation in the GI tract, as well as energy metabolism, by activating G-protein-coupled receptors on cells of the liver and GI tract [180].

### 11.2. A Role for S1P and Conjugated Bile Acids in Biliary Duct Cancers

There are some clear indications that bile acid accumulation indirectly facilitates bile duct proliferation, underlying biliary duct cancers (cholangiocarcinomas) [181], but the underlying mechanisms of action are currently unclear. There are a few emerging studies that demonstrate a strong connection between conjugated bile acid and activation of the S1P signalling pathways to inflammation and increases in hepatic carcinogenic risk, with SphK1 and SphK2 playing different roles in this process.

In 2012, Studer et al. [182] demonstrated a link between conjugated bile acids and activation of S1PR2 in the regulation of kinase (ERK)1/2 and protein kinase B (AKT) signalling pathways in primary hepatocytes [182]. However, the physiological role of bile acid activation of ERK1/2 on hepatic liver metabolism was not shown. Nagahashi et al. [166] demonstrated conjugated bile acid produced after eating a meal activated S1PR2. In turn, S1PR2 activated intracellular signalling pathways, which activated nuclear SphK2 and the catalysation of nuclear S1P. They also demonstrated that an increase in nuclear S1P inhibited specific histone deacetylases involved in the regulation of genes involved in, and maintaining, nutrient metabolism [166]. Nagahashi et al. [166] suggest activation of this S1PR2-SphK2-nuclear S1P as a mechanism to regulate hepatic lipid metabolism and to maintain nutrient homeostasis.

Discovery of the changing dynamics of sphingolipids in a small cohort of BTC patient samples (15 patients), using lipidomic analysis, revealed that major metabolic pathways for ceramide synthesis are enhanced in BTC compared to normal biliary tract tissue [175]. Whilst ceramide per se did not increase in these BTC patient samples, the levels of S1P and SphK1 were significantly elevated and SphK1 correlated positively with lymphatic metastasis-related substrate. In the same study, Hirose et al. [175], using immunohistochemistry in patient gallbladder tissue, demonstrated that high expression of the activated SphK1 (phosphorylated-SphK1), but not SphK2, was elevated in BTC with lymph node metastasis (metastatic GBCs) but not associated with lymphangiogenesis. In other studies, alterations in bile acid function contributed to malignant transformation of the cholangiocytes, the epithelial cells that line the bile ducts and, in part, increases in the SphK1/S1P pathway were shown to facilitate this transformation [166,183]. The overall conclusion supports the hypothesis that increased levels of S1P, resulting in changes in the S1P gradient between lymphoid organs and circulatory fluids in BTCs and GBCs, are associated with, and contribute to, lymphatic metastasis. This speculatively places SphK1 and S1P as potential diagnostic markers and targets for invasive cholangiocarcinomas. Although GBCs are usually studied jointly with BTCs, there was one study that found S1PR1 expression to be elevated in GBCs and associated with metastatic progression [184].

In summary, there is emerging evidence to form a positive connection between overly produced conjugated bile acids and dysregulation of SphK/S1P signalling in the development and progression of BTCs; however, the underlying mechanisms are still being uncovered.

## 12. S1P in Pancreatic Function and Cancer

Pancreatic ductal cancers are relatively uncommon, difficult to detect, and frequently diagnosed at an advanced stage with low survival rates due to reduced effective treatment options [185,186]. Chronic inflammation, attributable to obesity, diabetes, diet, and pancreatitis, usually precedes pancreatic cancers [187,188]. Extensive studies have focused on SphK/S1P/S1PR and insulin signalling in pancreatic beta cells due to their importance in inflammation, insulin resistance, and diabetes [189,190]. More recently, SphK/S1P/S1PR signalling modulators are being investigated for hard-to-treat and chemo-resistant pancreatic cancers to overcome intrinsic and acquired drug resistance [191,192,193,194]. Elevated SphK1 was found to be a characteristic of many pancreatic adenocarcinoma ductal lesions, with SphK1 thus being cited as a potential prognostic marker for pancreatic cancer [195]. The cellular ratio of ceramide and S1P was also cited as a critical biosensor of pancreatic cancer sensitivity to the chemotherapy agent gemcitabine [191]. Several studies, in vitro, and in vivo, are currently unravelling potential S1P pathways in pancreatic cancer development and how these pathways may contribute to resistant phenotypes [192,193,194]. One such pathway being extensively studied is conjugated bile acid (tauroursodeoxycholic acid (TUDCA))’s activation of S1PR2-ERK1/2/AKT and SphK2 due to its capacity to promote pancreatic survival [196]. TUDCA’s activation of S1PR2-ERK1/2/AKT/SphK2/S1P is linked to chemo-drug resistance in pancreatic cells [193].

Although SphK/S1P modulators may be promising treatment options, a potential problem with targeting SphK/S1P in pancreatic cancer is the fact that SphK1/S1P is necessary for the survival of the pancreatic cells; loss of SphK1 is linked to pancreatic beta-cell death and predisposition of diet-induced diabetes types 1 and 2 [197,198,199]. The challenge of using SphK/S1P modulators for pancreatic cancer treatment is balancing cancer cell apoptosis with the *β*-cell survival necessary for insulin production [29].

## 13. S1P and SphK Modulators in Clinical Trials and in the Clinic for GI Tract Cancers

In September 2010, FTY720 (brand name Gilenya^TM^) was the first-in-class oral bioavailable S1P immunomodulator approved by the FDA for clinical use as an immune modulator for multiple sclerosis patients. FTY720 is a non-selective S1PR modulator that blocks S1PR1, -3, -4, and -5, but not S1PR2 (Figure 2). Included in the known serious long-term side effects of FTY720 are bradycardia, recurrence, basal-cell carcinoma, migraines, increases in infections (especially fungal infections), and macular oedema. An important consideration for FTY720 therapy is the potential for increased bleeding, by blocking S1PR1; S1PR1 is critical for maintaining the blood vessel barrier integrity [152]. Thus, more targeted S1PR therapy has the potential to minimise adverse side effects. In recent years, the newer generation of S1P modulators are being developed to selectively target more specific S1PRs for more effective precision therapy with fewer side effects (Table 3). As mentioned earlier, the exciting news in the fight against chronic inflammation is the FDA approval of the S1P modulator, ozanimod (Zeposia^TM^), for UC, which selectively targets S1PR1 and -5. However the safety profile of ozanimod is unique to each individual and, hence, extensive safety, dosage, and pharmacokinetics factors are important considerations prior to treatment [153]. In June 2021, Arena Pharmaceuticals (San Diego, CA, USA) were granted an Orphan Drug designation by the FDA for etrasimod (targets S1PR1, -4, and -5) for the treatment of a rare eosinophilic oesophagitis disease, a chronic immune system disease where eosinophils (a type of white blood cell) build up in the lining and block the oesophagus. Although there is currently no direct evidence specifically linking eosinophilic oesophagitis to cancer, chronic inflammation of the oesophagus is a well-documented risk factor for oesophageal cancers [200]. Arena have also developed the use of etrasimod for IBDs and etrasimod is currently in Phase-III clinical trials for IBDs (Table 3). There will be much interest in discovering the difference in efficacy between ozanimod, which blocks S1PR1 and 5, and etrasimod, which blocks S1PR1, -4, and -5. The main goal is to use S1P modulators as a first line therapy with higher efficacy and with a good safety profile and few side effects. As new generations of selective S1PR and SphK agonists and antagonists are developed, hopefully, this will lead to a refined approach to treatment with better safety. Overall, the targeting of S1PRs and SphK provide significant therapeutic opportunities for treating chronic inflammation of the digestive system, which underpins many GI tract cancers.

## 14. Conclusions and Further Perspective

The infamous saying, you are what you eat, derived from a quote from Anthelme Brillat-Savarin in 1826, ‘Dis-moi ce que tu manges, je te dirai ce que tu es’, still holds true today. Being overweight/obese and a HFD are associated with compromised immune functions leading to chronic low-grade inflammation of the digestive system. Chronic inflammation creates an environment that is conducive to a greater risk of GI cancer development. Integrity of the sphingolipid metabolism is central to the regulation of inflammation and physiological homeostasis. There is a positive relationship between a HFD, with an oversupply of sphingolipid metabolites significantly altering cellular metabolism and altered downstream sphingolipid-mediated mucosal immune signalling pathways. Hence, nutrition can be a powerful tool to counteract chronic inflammation.

The bioactive sphingolipid metabolite S1P is one of the key sphingolipids involved in the GALT innate and adaptive immune responses, involved in trafficking, differentiation, and activation of immune cell effector functions. Over the past three decades, S1P and the S1P receptors have been identified as key players in maintaining immune homeostasis and in the pathophysiological processes of inflammatory diseases. High expression of S1P is associated with cancer-associated self-survival, mitogenesis, proliferation, angiogenesis, invasion, migration, and importantly, changes in the inflammatory response, thereby increasing the risk of many chronic disease states.

Here, we briefly discussed the direct and indirect effects of excessive dietary sphingolipid metabolites and the emerging potential of the gut biome in disturbing the immune homeostasis of the digestive system, maintaining a chronic low-grade inflammation, and predisposing the gut epithelial and associated digestive organs to carcinogenesis. We provided contemporary information on S1P/SIPRs’ systemic involvement in chronic inflammatory cancers of the digestive system and how bioactive sphingolipid S1P receptor modulators could be part of a meaningful novel therapeutic regime to counter for the unmet needs of hard-to-treat GI inflammatory-based cancers.

We now have several promising S1PR-based immunomodulators in clinical trials and in the clinic to treat GI inflammatory diseases. In the past year, ozanimod (Zeposia^TM^), which specifically targets S1PR1 and S1PR5, has FDA approval for treatment of UC, a condition that increases the risk of colorectal cancer. Etrasimod, which targets S1PR1, S1PR4, and S1PR5, has FDA orphan drug status to treat a rare chronic inflammatory disease eosinophilic oesophagitis and is currently in phase-III clinical trials for the treatment of IBDs. Whether these S1PR modulators prove to have long term benefits in reducing risk of GI cancers is yet to be determined. However, the results to date show that S1PR modulators have a promising outlook in the future as immunomodulators for the prevention of chronic inflammatory diseases and the treatment of GI inflammatory cancers.

## Figures and Tables

**Table 1:** Cancers of the digestive system: patient incidence and mortality estimates worldwide for the year 2020 and projected increase in 2040 [1].

Cancer Type	Incidence/Mortality (Year 2020)	Incidence/Mortality (Year 2040)	% Increase 2020–40 Incidence/Mortality
Lip/oral cavity	377,713/177,757	545,396/275,164	+54/+55
Salivary glands	53,583/22,778	82,039/37,114	+69/+65
Oropharynx	98,412/48,143	142,797/80,858	+65/+61
Larynx	184,615/99,840	285,720/158,846	+61/+60
Hypopharynx	84,254/38,599	N/A	N/A
Nasopharynx	133,354/80,008	N/A	N/A
Oesophagus	604,100/544,076	953,329/867,386	+63/+63
Stomach	1,089,103/768,793	1,758,810/1,366,121	+62/+56
Colon	1,148,515/576,858	1,919,534/1,016,453	+60/+57
Rectum	732,210/339,022	1,173,707/547,565	+62/+62
Anus	50,865/19,293	78,106/32,086	+65/+60
Liver	905,677/830,180	781,631/1,284,252	−16/+65
Gallbladder	115,949/84,695	385,005/295,368	+30/+29
Pancreas	495,773/466,003	815,276/777,423	+61/+60

Statistics from Global Cancer Observatory website, available online: https://gco.iarc.fr/today/online-analysis, accessed on 1 July 2021.

**Table 2:** The S1P receptors expression on immune cell subtypes and proposed S1P-S1PR functions.

S1PR	Innate Immune Subtype	Proposed Function
S1PR1	Macrophages Dendritic cells Eosinophils and mast cells Neutrophils Monocytes Natural killer cells T and B-lymphocytes	Recruitment, anti-inflammatory response, apoptosis Trafficking, inhibition of IFN-a secretion Recruitment Recruitment Trafficking Lymph node egress Guides lymphocytes out of lymphoid organs into circulatory fluids
S1PR2	Macrophages Dendritic cells Eosinophils and mast cells Monocytes	Enhanced antibody mediated phagocytosis Not expressed Degranulation Expressed but function not described Regulation of migration
S1PR3	Macrophages Dendritic cells Eosinophils and mast cells Neutrophils Monocytes	Recruitment and bacteria killing Maturation, promotion of Th1 response, Suppression of Treg Recruitment Recruitment Circulation and possible recruitment
S1PR4	Macrophages Dendritic cells Eosinophils and mast cells Neutrophils Monocytes	Expressed but function not described Plasmacytoid differentiation, inhibition of IFN-a secretion Expressed but function not described Recruitment Expressed, potential modulation of neutrophil migration Cell migration
S1PR5	Eosinophils and mast cells Monocytes Natural killer cells	Expressed but function not described Patrolling monocyte trafficking Bone marrow egress Cell migration

**Table 3:** Clinical trials using S1PR and SphK modulators for the treatment of inflammatory gastric-intestinal tract- and organ-related disorders.

S1P Modulator	S1PR Target	Disease	Clinical Trial	NCT Number (ClinicalTrials.gov)	Status *
Amiselimod	S1P1,4,5	Crohn’s disease	Phase II	NCT02389790	C
				NCT02378688	C
Etrasimod	S1P1,4,5	Ulcerative colitis Crohn’s disease Primary biliary cholangitis Ulcerative colitis Ulcerative colitis Ulcerative colitis	Phase II Phase III Phase III Phase III Phase III	NCT02447302NCT03139032 NCT02536404 NCT03155932 NCT03996369 NCT03945188 NCT03950232	C A T R A A A
RPC1063	S1P1,5	Crohn’s disease		NCT02531113	C [154]
Ozanimod # (RPC1063)	S1P1,5	Ulcerative colitis, Crohn’s disease	Phase III	NCT02531126	R
				NCT03467958	R
				NCT02435992	C [155,156]
				NCT03464097	R
				NCT03440385	R
				NCT03440372	R
				NCT03915769	R
GSK2018682	S1P1,5	Healthy volunteers	Phase I	NCT01466322	C
				NCT01387217	C
				NCT01431937	C
ASP4058	S1P1,5	Healthy volunteers	Phase I	NCT0199866	C
Mocravimod	S1P1(4,5?)	Ulcerative colitis	Phase II	NCT01375179	T
Ceralifimod	S1P1,5 (4?)	Ulcerative colitis, Crohn’s disease	Phase II	NCT02531126	R
				NCT02435992	R
				NCT03467958	R
SphK inhibitors	SphK target	Disease	Clinical trial	NCT number	Status *
ABC294640	SphK2	Pancreatic cancer	Phase I	NCT01488513	C

* A = active, C = completed, R = recruiting, T = terminated (adapted from [62] and NIH website). # Ozanimod (Zeposia^TM^) is the first S1P receptor modulator approved by the FDA for UC.
